# The contextual self: object ownership modulates neural encoding across peripersonal and extrapersonal spaces

**DOI:** 10.1038/s41598-026-44438-z

**Published:** 2026-03-24

**Authors:** L. Lenglart, Y. Coello, A. Sampaio

**Affiliations:** 1 UMR 9193 - SCALab - Sciences Cognitives et Sciences Affectives, Univ. Lille, CNRS, 59000 Lille, France; 2 FR 2052 - SCV - Sciences et Cultures du Visuel, Univ. Lille, CNRS, 59200 Tourcoing, France; 3 Psychological Neuroscience Lab, Psychology Research Centre (CIPsi), School of Psychology, University of Minho, Braga, Portugal

**Keywords:** Peripersonal space, Ownership, fMRI, Spatial perception, Self-relevance, Neuroscience, Psychology, Psychology

## Abstract

A crucial aspect of interacting with objects in our environment lies in determining whether they are located within peripersonal space (PPS) - where immediate action is possible- or extrapersonal space (EPS), where objects are out of reach. Importantly, social context often prompts additional consideration of object ownership, which may interfere with spatial localization. Although previous research has demonstrated that both spatial and social contexts influence object processing, the neural networks subtending their interaction remains largely unexplored. To address this issue, the present fMRI study investigated the neural correlates of object ownership as a function of spatial location (PPS vs EPS). While facing a virtual character, 22 participants judged the reachability of self-owned or other-owned objects placed at varying distances. Results showed that objects located in the PPS activated parietal regions implicated in the sensorimotor coding of near-space stimuli, with enhanced parietal responses observed for self-owned objects, particularly in the right hemisphere. Conversely, objects in EPS engaged prefrontal regions, especially when they were self-owned. Multivariate analyses further revealed that both the ventromedial prefrontal cortex (vmPFC) and dorsomedial prefrontal cortex (dmPFC) reliably distinguished self-owned objects, with the vmPFC selectively encoding self-ownership in the PPS. These findings highlight how spatial and social dimensions jointly shape object representations in the brain, with self-relevance modulating action-related processing even in tasks focusing on spatial processing.

**Supplementary Information:**

The online version contains supplementary material available at 10.1038/s41598-026-44438-z.

## Introduction

Spatial perception is inherently non-uniform. Rather than representing space as homogeneous and continuous, the brain constructs nested spatial representations, each serving distinct functional purposes. For instance, peripersonal space (PPS) refers to the region of space immediately surrounding the body that is most directly involved in interactions with nearby objects and individuals, and is supported by specialized neurocognitive mechanisms that integrate multisensory information^1^. In an action-oriented context, PPS can be functionally distinguished from extrapersonal space (EPS; i.e., the space beyond PPS) as the space within one’s immediate reach^2^. In this sense, PPS is uniquely suited for guiding actions by integrating multisensory inputs in a self-centered frame of reference^1,2^, and is defined by its behavioral relevance for action rather than by fixed metric distances^3^. Accordingly, PPS is a flexible and context-sensitive representation that adapts to changing bodily and environmental constraints. For example, the boundaries of PPS expand following tool use, as the body schema incorporates the tool as an extension the limb wielding it^4–7^, or contract when bodily movements are restricted^8,9^. This plasticity allows the brain to continuously recalibrate spatial representations, thereby supporting efficient motor responses and optimizing interactions with the environment^3,10,11^.

Neurophysiological studies have established that PPS representation relies on a distributed fronto-parietal network specialized for multisensory integration and action preparation^12–14^. In this respect, seminal work in non-human primates identified bimodal neurons in the ventral premotor cortex (areas F4 and F5) and in the intraparietal sulcus (areas VIP and AIP) that respond to both tactile inputs and visual or auditory stimuli presented near the body^13,15–17^. In humans, convergent evidence from neuroimaging and lesion studies revealed a functionally homologous network comprising the inferior parietal lobule (IPL), intraparietal sulcus (IPS), and the dorsal and ventral premotor cortex (PMC), selectively activated during near-space processing^14,18,19^. Critically, this core PPS network does not operate in isolation but functions as part of a flexible, context-sensitive system: its recruitment is modulated via coordinated connections with other neural systems, depending on task demands, body posture, and environmental context^12^.

Over the past years, PPS has also been conceptualized as a dynamic interface for social interaction. Behavioral studies have shown that during in cooperative tasks, individuals adapt their PPS representation to include their partners’ space, thereby facilitating coordinated action^20,21^. This remapping is supported by the construction of a representation of others’ PPS, allowing for anticipated motor adjustments^22,23^. Neuroimaging evidence indicated that fronto-parietal regions involved in the PPS representation -including the premotor and parietal cortices- exhibited activity patterns mirroring self-related processing when others are present^24–26^. These findings challenged strictly self-centered models of PPS, demonstrating that PPS representation also incorporate interpersonal information to support action coordination and social interaction^10^.

A key dimension through which social context influences spatial representations is the attribution of object ownership. When an object is within our reach, implicit social norms can constrain our interaction with it, as objects within our PPS may belong to us or to others, depending on the context. This creates a situation where individuals simultaneously feel the urge to engage with objects in their immediate space, while also experiencing a reluctance to interact with items that belong to others^27–30^, due to the widely accepted norm that we do not spontaneously engage with others’ belongings without explicit permission. Indeed, individuals are motivated to maximize personal benefits while simultaneously minimizing social risks^31^. Thus, recognizing the ownership of objects in our environment is crucial for selecting and guiding appropriate interactions with both the objects and the individuals involved^28,30^.

Beyond its role in social organization, object ownership also influences the idiosyncratic relevance of objects. Through enhanced self-relevance, items perceived as belonging to oneself acquire a privileged status in cognitive processing, a phenomenon termed “self-prioritization”^32^. This effect includes a range of advantages, such as enhanced attentional focus and improved memory encoding through preferential processing (see^32^ for a review). Neuroimaging studies have consistently linked this self-prioritization to changes in the activation of cortical midline structures, particularly the ventromedial (vmPFC) and dorsomedial (dmPFC) prefrontal cortices, which are recognized as key regions supporting self-referential cognitive functions^33–36^. Importantly, the implication of this neural regions extends from abstract self-referential information to self-owned objects: for instance, Turk et al.^37^ showed that the activity of the vmPFC and dmPFC was selectively modulated by object ownership, with the dmPFC being specifically engaged in the processing of self-owned objects. Similarly, Kim & Johnson^38^ proposed that the preferential activation of the mPFC provides evidence for the incorporation of self-owned objects into an “extended self”, referring to the integration of stimuli into one’s own representation of the self^39,40^. Interestingly, these regions are also involved in the broader processing of socially relevant information^41^, suggesting that ownership may serve as a bridge between self-related and socially contextualized cognition.

However, the extent to which this self-prioritization reflects automatic processing or varies according to context remains a topic of ongoing debate^42,43^. Recent studies, for instance, have challenged the assumption that self-relevant stimuli are invariably prioritized: when task demands do not explicitly involve self-referential processing, self-prioritization is often significantly attenuated^44^ or even absent^45,46^. Consistent with this interpretation, enhanced processing of self-owned objects has been observed specifically when those objects are located within PPS, rather than EPS^29,30,47^. However, the neurocognitive mechanisms through which contextual factors modulate the prioritization of self-relevant objects remain poorly understood. To address this gap, the present study investigated how object ownership (self- vs. other-owned) and spatial representation (PPS vs. EPS) interact at the neural level. Using functional magnetic resonance imaging (fMRI), we investigated the involvement of the fronto-parietal network, known for its role in PPS processing, alongside brain regions typically associated with self-referential cognition. To determine whether self- and other- owned objects recruited distinct brain networks depending on their spatial location (PPS vs. EPS), participants performed a reachability judgment task adapted from previous protocols^29,30^. During fMRI scanning, they estimated the reachability of cups attributed either to themselves or to another person.

This design enabled us to examine (I) whether reachability judgements for self- and other-owned objects recruited similar or distinct neural networks depending on spatial context, (II) whether ownership modulated activity within the core fronto-parietal PPS network, and (III), whether activation patterns within the medial prefrontal cortex distinguished between self- and other-owned objects as a function of their spatial location. Accordingly, we (I) conducted an exploratory whole-brain analysis to test for ownership-by-space interactions across the brain as a whole. For (II), if ownership is embodied within the sensorimotor system, as proposed by Constable et al.^27^, we predicted stronger activation for self- relative to other-owned objects within the fronto-parietal PPS network, specifically when objects were presented in the PPS. Finally, for (III), we considered two possibilities regarding medial prefrontal cortex involvement: if ownership constitutes a stable self-related feature, vmPFC activity should preferentially encode self-owned objects regardless of spatial location (with dmPFC encoding other-owned objects). By contrast, if ownership is context-dependent, vmPFC recruitment should emerge selectively for self-owned objects presented within the PPS.

## Results

### Whole-brain analysis

For each whole-brain analysis, voxel significance was thresholded at p < 0.001 (uncorrected^48^), with clusters considered as significant at p < 0.05 (FDR-corrected).

#### Main effect of Space

**Fig. 1:**
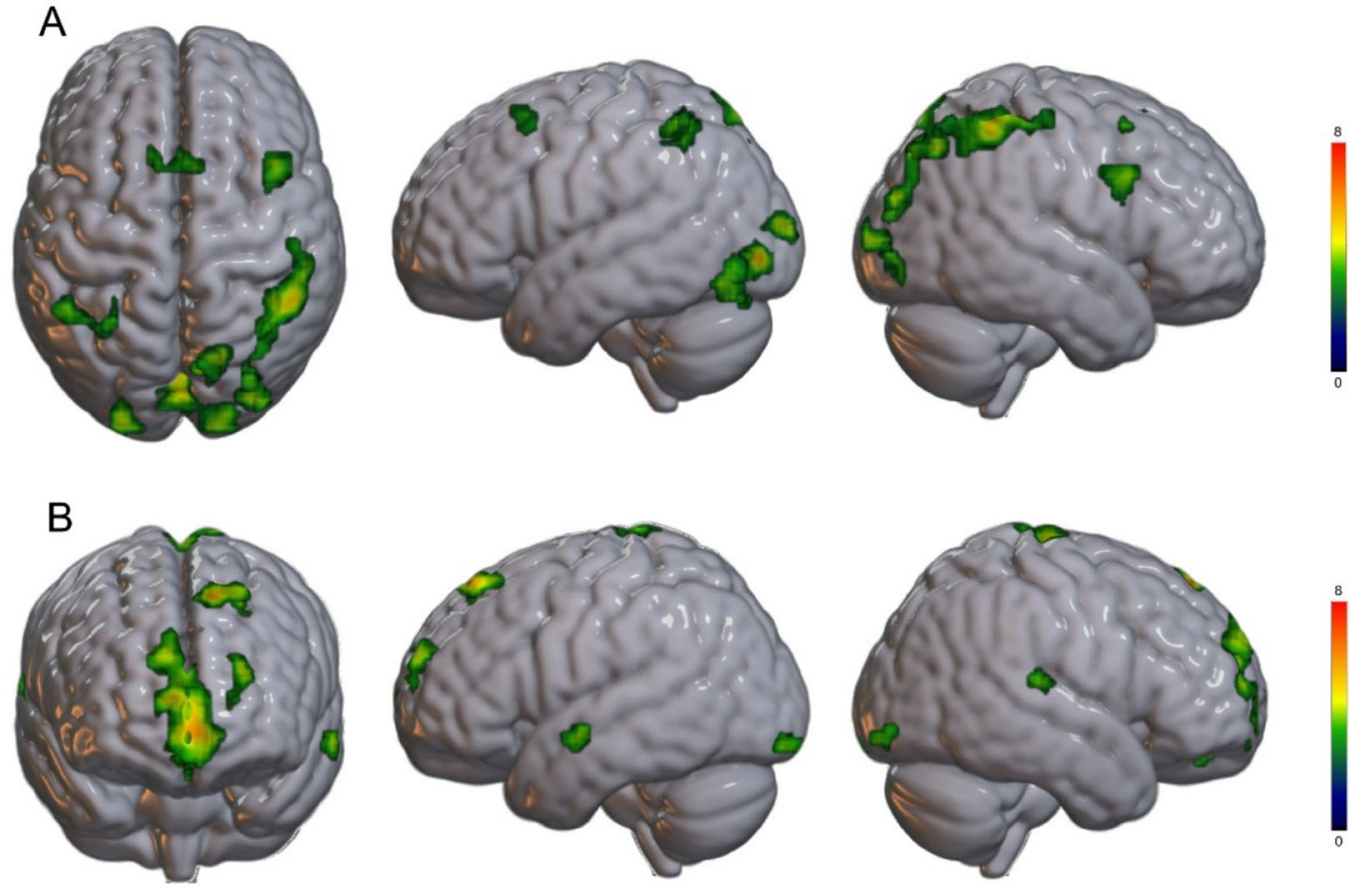
Brain areas more active for cups located in (A) PPS vs. EPS and (B) EPS vs. PPS, surviving at a statistical threshold of *p*_*uncorrected*_ <0.001 at the voxel level and *p*_FDR_<0.05 corrected at the cluster level.

The *t*-contrast examining the main effect of spatial location *(PPS > EPS)* revealed bilateral activations in the parietal cortex. In the left hemisphere, significant clusters emerged in the superior and inferior parietal lobules (SPL and IPL; peak MNI coordinates: -26, -53, 45; see Table 1 and Fig. 1a). In the right hemisphere, activations were observed in the SPL, IPL, and precentral gyrus (MNI: 49, -44, 54), partly corresponding to the anterior part of the intraparietal sulcus (aIPS), with an additional cluster extending to the precuneus and cuneus (MNI: 19, -68, 45).

**Table 1:** Brain regions showing significant activations for (a) the contrast between peripersonal space and extrapersonal space and (b) the contrast between extrapersonal and peripersonal space.

		Peak-level					Cluster-level		
Macroanatomical location	Microanatomical composition	x	y	z	*T*	*P*	Number of voxels	*P *_*uncorrected*_	*P *_*FDR*_
**PPS – EPS**									
Left and right calcarine sulcus, Lingual gyrus	hOc1 (V1) (37,3%), hOc2 (V2) (14,7%), hOc3v (V3v) (12,8%), hOc4v (V4v) (9.7%),	-8	-84	-3	7.48	0	303	0	0
		4	-84	-6	6.45	0			
		16	-99	6	6.26	0			
Right inferior parietal lobule, Superior parietal lobule, Postcentral gyrus	hIP3 (IPS) (12.7%), PFm (IPL) (9.2%), area 1 (7.9%), hIP2 (IPS) (7.5%)	49	-44	54	6.47	0	313	0	0
		40	-44	51	6.15	0			
		28	-87	24	6.01	0			
Right cuneus, Precuneus, Superior parietal lobule, Superior occipital gyrus	Area 7P (SPL) (41.1%), Area 7A (SPL) (4.0%)	19	-68	45	6	0	89	0	0
		7	-77	57	5.34	0			
		13	-68	66	4.13	0			
Left middle occipital gyrus, Superior occipital gyrus	hOc3d (V3d) (38%), hOc4p (22.8%), hOc4d (V3a) (16.8%), hOc1 (7.1%)	-23	-93	12	5.93	0	48	0.001	0.011
Right pars opercularis, Middle frontal gyrus, Pars triangularis	BA44	40	13	33	5.22	0	62	0	0.003
Left supplementary motor area, Right supplementary motor area	6mr, pre-SMA (37.4%)	7	13	54	5.04	0	45	0.001	0.016
		-8	10	57	4.92	0			
Left superior parietal lobule, Inferior parietal lobule	hIP3 (IPS) (26%), hIP2 (IPS) (14%), 7A (SPL) (10.3%), area PFm (IPL) (7.6%)	-26	-53	45	4.84	0	72	0	0.001
		-44	-47	51	4.83	0			
		-26	-53	57	4.3	0			
**EPS – PPS**									
Left superior frontal gyrus, Medial frontal gyrus	BA8	-11	38	54	8.13	0	91	0	0
		-23	32	57	5.83	0			
		-26	38	51	5.5	0			
Left frontal middle orbital, Frontal superior medial, Anterior cingulate cortex (pregenual) Right medial orbital cortex	p32 (29.2%), Fp2 (18.4%)	-5	56	-3	7.17	0	643	0	0
		7	56	12	6.45	0			
		-5	53	6	5.75	0			
Left paracentral lobule, SMA, Postcentral gyrus Right paracentral lobule	4a (22.7%), BA6	-3	-23	75	6.65	0	44	0.001	0.011
		-14	-32	78	5.35	0			
		7	-17	78	4.03	0			
Left superior frontal gyrus, Middle frontal gyrus	Fp1 (10.8%), BA10	-23	59	24	5.52	0	40	0.001	0.011
		-20	62	12	3.99	0			
Right temporal superior gyrus, Supramarginal gyrus	TE3 (20.2%), PFcm (IPL (17.4%), PF (IPL) (13.4%)	65	59	12	4.97	0	24	0.009	0.042
Left lingual gyrus, Occipital inferior gyrus, Cerebellum crus 1	hOc3v (72.7%), hOc2 (19.8%), hOc4v (7.1%)	-20	-93	-15	4.89	0	39	0.001	0.011
Left temporal middle gyrus, Temporal superior gyrus	TE3 (24.3%), BA21	-63	-8	-9	4.87	0	29	0.005	0.029
Right lingual gyrus, Inferior occipital gyrus, Calcarine sulcus	hOc3v (75.6%), hOc2 (23.8%)	19	-93	12	4.73	0	24	0.009	0.042

x, y, z = peak coordinates (MNI); T = t-statistic.

The reverse contrast *(EPS > PPS)* revealed widespread frontal activations. These included a cluster encompassing the left superior and medial frontal gyri (MNI: -17, 62, 15; see Table 1 and Fig. 1b), as well as bilateral activation in medial orbitofrontal regions, including the frontal medial orbital and superior medial cortices.

#### Main effects of ownership

No significant activation was observed for the main effect of ownership, as tested by the contrasts *Self > Other* and *Other > Self*, at the whole-brain level.

#### Space × Ownership interaction

To assess how ownership and spatial location jointly modulate neural activity, we additionally conducted a whole-brain analysis of the interaction between the four conditions. This analysis revealed two significant clusters in the frontal cortex for the contrast *Self > Other* in the PPS compared to EPS (MNI: -23, 59, 6 and -20, 47, 45; see Table 2). These results suggest that frontal regions are sensitive to the congruency or contextual alignment between ownership and spatial proximity.

**Table 2:** Brain regions showing significant activations for the contrast between all factors.

		Peak-level					Cluster-level		
Macroanatomical location	Microanatomical composition	x	y	z	*F*	*P*	Number of voxels	*P *_*uncorrected*_	*P *_*FDR*_
Interaction									
Left superior frontal gyrus, middle frontal gyrus, medial superior frontal gyrus	BA10	−23	59	6	37.8	0	44	0	0.003
		−23	56	18	25.3	0			
Left superior frontal gyrus	Area Fp1(29.5%), BA8	−20	47	45	27.8	0	46	0	0.003
		−23	35	48	21.3	0			
		−23	26	45	18.1	0			

x, y, z = peak coordinates (MNI); F = f-statistic.

**Fig. 2:**
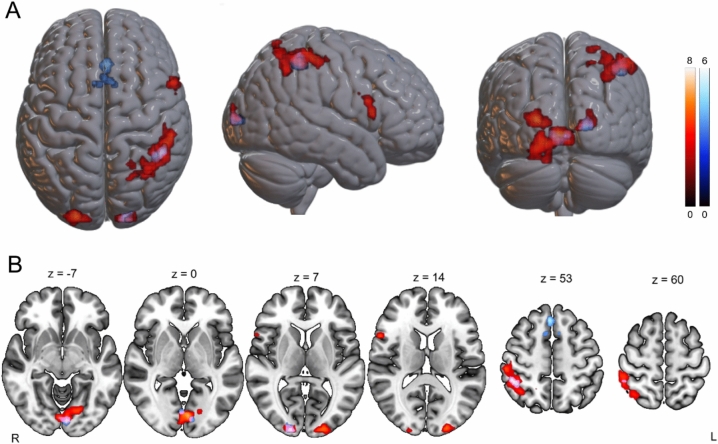
Brain areas more active for cups located in the PPS vs. EPS for self-owned objects (red) and other-owned objects (blue), surviving at a statistical threshold of *p*_*uncorrected*_ <0.001 at the voxel level and *p*_FDR_<0.05 corrected at the cluster level.

To better identify brain regions involved in processing objects in near versus far space for each ownership condition, we separately analyzed the contrasts comparing PPS to EPS and EPS to PPS for self-owned and other-owned objects. For self-owned objects, the contrast *PPS > EPS* revealed a large right-lateralized parietal cluster extending along the anterior-posterior axis of the intraparietal sulcus (IPS), encompassing the IPL, SPL, supramarginal gyrus, and precentral gyrus (MNI: 55, -32, 57; Table 3 and Fig. 2). Additional clusters were found in the right inferior frontal gyrus (rIFG; opercular and triangular parts; MNI: 59, 10, 21) and anterior precentral gyrus (MNI: 55, 16, 12). Bilateral occipital activations were observed in the calcarine sulcus and superior occipital gyrus, with further involvement of the right cuneus and left lingual gyrus.

**Table 3:** Brain regions showing significant activations for (a) the contrast between peripersonal space and extrapersonal space for self-owned objects and (b) the contrast between peripersonal space and extrapersonal space for other-owned objects.

		Peak-level					Cluster-level		
Macroanatomical location	Microanatomical composition	x	y	z	*T*	*P*	Number of voxels	*P *_*uncorrected*_	*P *_*FDR*_
**PPS self – EPS self**									
Left calcarine sulcus, Lingual gyrus	hOc1 (V1) (30.1%), hOc3v (V3v) (20.2%), hOc4v (V4v) (13.8%), hOc2 (V2) (11.6%)	-5	-84	-3	8.22	0	227	0	0
		-23	-71	-12	8.82	0			
		-14	-74	-9	8.63	0			
Left middle occipital gyrus, Superior occipital gyrus	hOc3d (v3d) (37.3%), hOc4lp (25.2%), hOc4d (V3A) (14.4%), hOc1 (V1) (9.0%)	-23	-96	9	7.33	0	24	0	0.001
Right postcentral gyrus, Inferior parietal lobule, Superior parietal lobule, Supramarginal gyrus	hIP3 (IPS) (14.7%), 7A (SPL) (11.5%), PFm (IPL) (10.1%), Area 1 (7.4%), Area hIP2 (IPS) (7.2%)	55	-32	57	6.3	0	246	0	0
		40	-41	51	5.37	0			
		37	-56	48	5.1	0			
Right inferior frontal operculum, Precentral gyrus, Pars triangularis	Area 44 (71.9%)	59	10	21	5.97	0	41	0.001	0.009
		55	16	12	5.82	0			
Right calcarine sulcus, Cuneus, Superior occipital gyrus	hOc1 (v1) (43.5%) hOc2 (v2) (24.0%) hOc3d (V3d) (15.6%) hOc3v (V3v) (3.4%)	13	-99	6	5.67	0	36	0.002	0.013
		22	-99	15	4.48	0			
**PPS other – EPS other**									
Left and right medial superior frontal gyrus, Supplementary motor area	Area 6mr / preSMA (10.1%), BA8, BA6	4	29	51	6.3	0	60	0	0.005
		7	13	51	4.12	0			
		-8	13	51	3.98	0			
Right inferior parietal lobule, Superior parietal lobule, Supramarginal gyrus	hIP3 (IPS) (35.0%), hIP2 (IPS) (32.0%), PFm (IPL) (23.1%), hIP1 (IPS) (2.7%)	46	-44	51	5.72	0	42	0.001	0.01
Right calcarine sulcus, Cuneus, Superior occipital gyrus.	hOC1 (V1) (57.2%), hOc3d (V3d) (6.5%), hOc2 (V2) (5.7%)	16	-99	6	5.53	0	29	0.004	0.036
Left calcarine sulcus, Lingual gyrus, Middle occipital gyrus	hOc1 (V1) (69.2%), hOc2 (V2) (18.7%)	-8	-87	-3	5.44	0	45	0.001	0.01
		1	-74	0	4.07				

x, y, z = peak coordinates (MNI); T = t-statistic.

For other-owned objects, the *PPS > EPS* contrast revealed right-lateralized activations in the IPL, SPL, and supramarginal gyrus (MNI: 46, -44, 51; see Table 3 and Fig. 2), though the cluster was smaller than that observed for self-owned objects. Occipital activation patterns resembled those seen in the self-owned condition, with bilateral calcarine activation, and additional clusters in the left lingual and middle occipital gyri, and right cuneus and superior occipital gyrus.

**Fig. 3:**
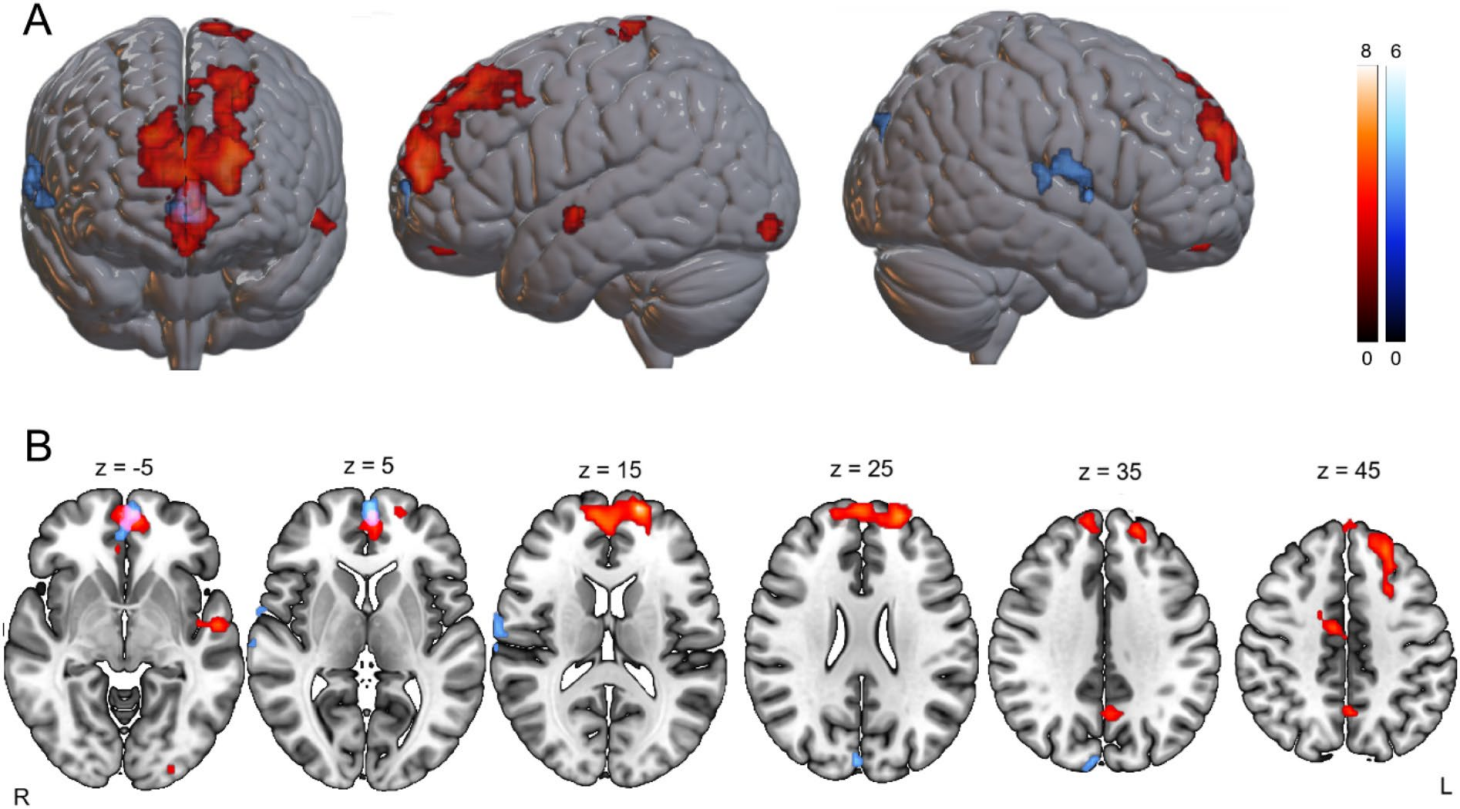
Brain areas more active for cups located in EPS vs. PPS for self-owned objects (red) and other-owned objects (blue), surviving at a statistical threshold of *p*_*uncorrected*_ <0.001 at the voxel level and *p*_FDR_<0.05 corrected at the cluster level.

The *EPS > PPS* contrast for self-owned objects revealed a large frontal cluster involving the left superior and medial frontal gyri, encompassing regions of the vmPFC, dmPFC, and dorsal anterior cingulate cortex (dACC; MNI: -17, 62, 15; see Table 4 and Fig. 3). Additional activations were observed in the right posterior superior temporal sulcus (pSTS), right SMA, bilateral midcingulate cortex, left paracentral lobule, and left postcentral gyrus. Posterior activations included the precuneus and left inferior occipital and lingual gyri.

**Table 4:** Brain regions showing significant activations for (a) the contrast between extrapersonal space and peripersonal space for self-owned objects and (b) the contrast between extrapersonal space and peripersonal space for other-owned objects.

		Peak-level					Cluster-level				
Macroanatomical location	Microanatomical composition	x	y	z	*T*	*P*	Number of voxels	*P *_*uncorrected*_			*P *_*FDR*_
**EPS self – PPS self**											
Left superior frontal gyrus, Medial superior frontal gyrus	Area Fp1 (30.6%) BA10, BA8, BA9	-17	62	15	7.62	0	933		0	0	
		-20	56	21	6.61	0					
		-23	38	48	6.54	0					
Left superior temporal gyrus, Middle temporal gyrus, Rolandic operculum	Area TE3 (8,4%) BA22, BA21	-57	-8	-3	5.79	0	39		0.001	0.015	
		-44	-5	-9	4.75						
		-66	-8	-9	4.72						
Left and right midcingulate cortex, Right supplementary motor area	Area 6mc / SMA (15,3%) BA24	7	-8	45	5.1	0	31		0.003	0.019	
		16	-2	45	4.09						
Left paracentral lobule, Postcentral gyrus	Area 4a (12%), Area 5 L (4,8%) BA2, BA4	-14	-32	78	5.02	0	33		0.003	0.018	
		-26	-26	75	3.84						
Left precuneus, Right precuneus	7A (6,2%) BA7	-2	-59	45	4.99	0	39		0.001	0.015	
		-5	-59	36	4.47						
Left lingual gyrus, Inferior occipital gyrus	hPc3V (V3v), (75,8%), hOc4lp (13,2%), hOc2 (V2) (9,2%)	-17	-93	-15	4.66	0	36		0.002	0,16	
**EPS other – PPS other**											
Left superior frontal gyrus, Medial superior frontal gyrus, Orbital gyrus, Pregenual anterior cingulate cortex	Area Fp2 (42.8%), Area p32 (14.9%)	-5	59	0	5.22	0	109		0	0	
		-2	65	6	5.18	0					
		1	47	-3	4.34	0					
Right superior temporal gyrus, rolandic operculum	Area OP4 (PV) (38.2%), Area TE3 (22.5%), Area TE 1,2 (2.8%),	68	-20	9	5.09	0	62		0	0.001	
		65	1	0	4.26	0					
		68	-8	15	4.22	0					
Left and right cuneus	Area hOc3d (V3d) (41.9%), Area hOc4d (V3A) (22.5%), Area hOc2 (V2) (12.6%)	7	-87	33	4.72	0	35		0.002	0.017	

x, y, z = peak coordinates (MNI); T = t-statistic.

For other-owned objects, *EPS > PPS* yielded a smaller but overlapping cluster in frontal regions. This included the left superior and medial frontal gyri, orbital gyrus, and pregenual anterior cingulate cortex, extending into the superior vmPFC and ventral ACC (MNI: -5, 59, 0; see Table 4 and Fig. 3).

### ROI analysis

To assess ownership-related modulation within the PPS and EPS, voxel significance was thresholded at *p* < 0.005 (uncorrected^49^), with clusters considered significant at *p* < 0.05 (FDR-corrected), in line with previous studies examining activations of the fronto-parietal PPS core network.

**Fig. 4:**
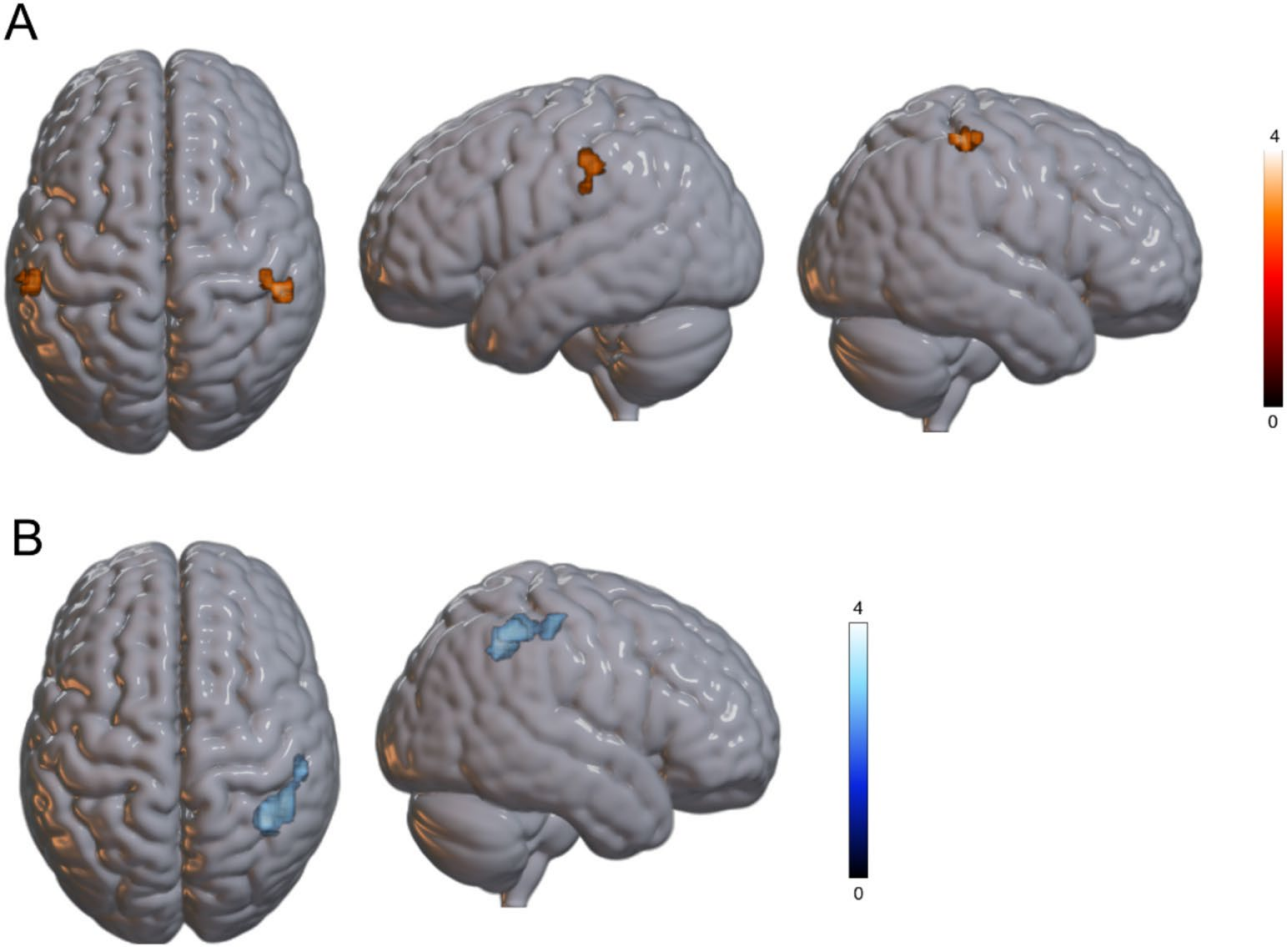
Brain areas more active for (**A**) self- vs. other-owned cups in the PPS and (**B**) other vs. self-owned cups in EPS, surviving at a statistical threshold of *p*_*uncorrected*_ <0.001 at the voxel level and *p*_FDR_<0.05 corrected at the cluster level.

When analyses were constrained to the fronto-parietal core PPS bilateral network, the contrast *Self > Other* in the PPS revealed significant bilateral parietal activations. On the right hemisphere, activations were found in the IPL, SPL, and precentral gyrus (MNI: 52, -35, 57; see Table 5 and Fig. 4), while on the left hemisphere, activation encompassed the IPL, supramarginal gyrus, and postcentral gyrus (MNI: -57, -26, 45). No significant activations were observed for the reverse contrast (*Other > Self*) within PPS.

**Table 5:** Brain regions showing significant activations for (a) the contrast between self-owned and other-owned objects in peripersonal space and (b) the contrast between other-owned and self-owned objects in extrapersonal space for other-owned objects, restricted to a ROI composed of the fronto-parietal network subtending processing in the PPS (Grivaz et al., 2017).

		Peak-level					Cluster-level		
Macroanatomical location	Microanatomical composition	x	y	z	*T*	*P*	Number of voxels	*P *_*uncorrected*_	*P *_*FDR*_
***PPS self > PPS other***									
Right inferior parietal lobule, Superior parietal lobule, Postcentral gyrus	Area 1 (39.5%), Area 2 (14.3%), Area of (ipl) (10.2%)	52	-35	57	4.01	0	36	0.017	0.047
Left inferior parietal lobule, Supramarginal gyrus, Postcentral gyrus	PFt (IPL) (78.5%), PFop (IPL) (10.6%), Area 2 (6.5%)	-57	-26	45	3.52	0	30	0.026	0.047
***EPS other > EPS self***									
Right angular gyrus, Supramarginal gyrus	hIP3 (IPS) (24.5%), hIP2 (IPS) (18.9%), hIP1 (IPS) (17.0%), PFm (IPL) (14.1%)	37	-53	45	3.63	0	116	0	0.002
		46	-41	51	3.45	0			
		34	-47	39	3.42	0			

x, y, z = peak coordinates (MNI); T = t-statistic.

In the EPS, the contrast *Self > Other* did not reveal any significant clusters of activation. In contrast, the reverse contrast *Other > Self*-identified a significant right-lateralized cluster encompassing the angular gyrus and the supramarginal gyrus (MNI: 37, –53, 45; see Table 5 and Fig. 4).

### Multivariate ROI-based analysis

**Fig. 5:**
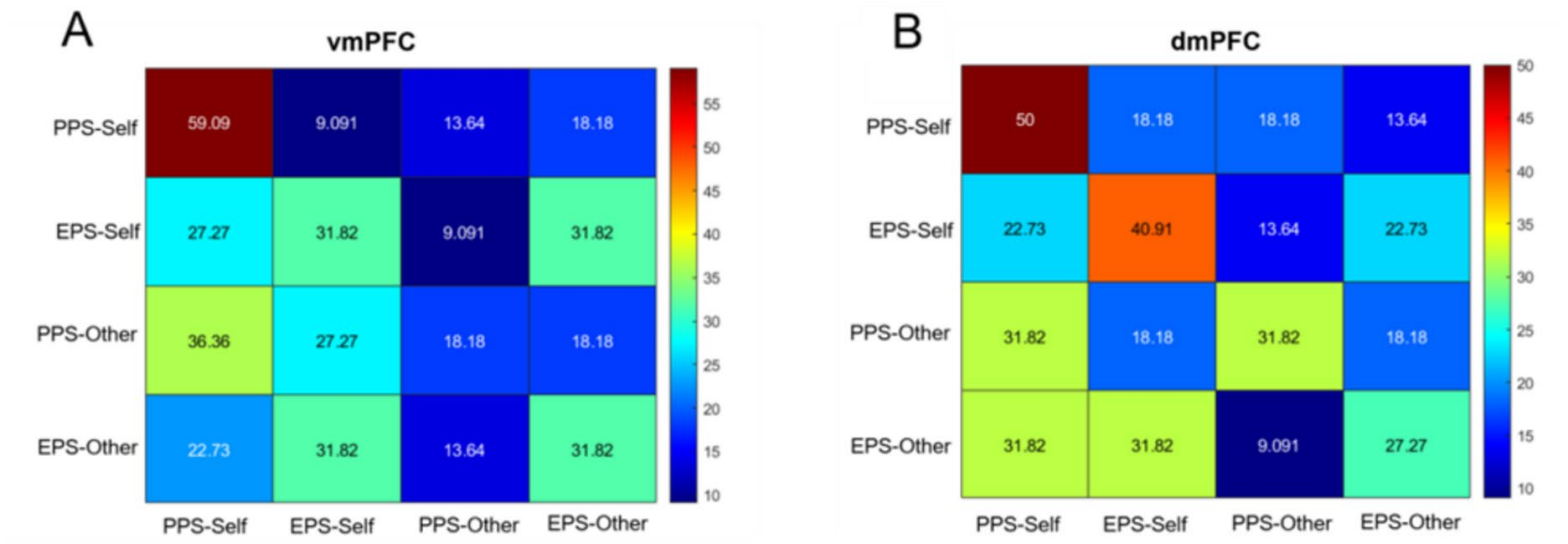
Confusion matrix of the multivariate pattern classification accuracy for the four experimental conditions in (**a**) the ventromedial prefrontal cortex and (**b**) the dorsomedial prefrontal cortex. Each row corresponds to the true experimental condition, and each column indicates the proportion of instances classified as each of the four possible conditions. Values represent the percentage of test instances assigned to each category, averaged across folds. Percentages of correctly classified conditions appear in the diagonal starting from the top-left corner of the matrix. Chance level is at 25%.

Multivariate pattern analysis (MVPA) revealed that activations patterns in the vmPFC carried significant information distinguishing self-owned objects presented in the PPS, with classification accuracy significantly above chance (59.09%; *p*_*uncorrected*_= .001, *p*_*Bonferroni*_ = 0.004, chance level at 25% for four conditions, see Fig. 5). None of the other conditions, including self- and other-owned objects in EPS and other-owned objects in the PPS, was classified above-chance in the vmPFC (all *p*_*uncorrected*_ > .15). Notably, in EPS, classification patterns for self- and other-owned objects were descriptively similar, suggesting that classification in this context was likely driven by spatial rather than ownership-related features. This pattern may reflect the encoding of general social or contextual salience rather than self-specific processing.

In the dmPFC, classification accuracy for self-owned objects in the PPS was also significantly above chance (classification accuracy = 50%, *p*_*uncorrected*_ =.01, *p*_*Bonferroni*_ = .04). A trend toward above-chance classification was observed for self-owned objects in EPS as well, though this effect did not survive correction for multiple comparison (classification accuracy = 50%, *p*_*uncorrected*_=.02, *p*_*Bonferroni*_ = .08). Classification performance for all other conditions in the dmPFC remained at chance level (all *p*_*uncorrected*_ >.14).

## Discussion

The present study investigated the neural mechanisms underlying reachability judgments within peripersonal space (PPS) and extrapersonal space (EPS), focusing on how object ownership modulates spatial encoding across these domains. Our findings reveal that spatial processing in the PPS and EPS engages distinct neural networks, aligning with prior research supporting a functional dissociation in the neural coding of near and far space. Crucially, this spatial encoding is not uniform across all stimuli: object ownership introduced socially grounded conceptual meaning that differentially influenced neural processing depending on the spatial context.

## Neural representation of PPS and EPS

Contrasting PPS with EPS conditions revealed significant activations within the parietal cortex, with peak responses localized in the rostral part of the IPL, in the SPL, and in the postcentral gyrus, partly overlapping with the aIPS. This pattern of activations is consistent with extensive literature linking these parietal regions to near-space coding^12–14,50^ and the representation of action-related information, including manipulable objects^51–53^. Although bilateral parietal engagement was observed, the predominantly right-lateralized activation pattern aligns with right-hemisphere dominance in visuospatial coding and body-space representation^54–59^. While this has been frequently observed in previous research^12^, no significant contralateral premotor activation was found in the present study. This lack of activation may be related to the reduced premotor recruitment often observed in tasks that do not require overt motor execution^14^, possibly due to rapid adaptation of premotor neurons to static visual stimuli^17^. Alternatively, it is also possible that sub-threshold premotor activity was insufficient to reach statistical significance, in line with the high incidence of type-II errors commonly reported in fMRI studies^60^.

Ownership exerted a significant influence on spatial encoding in the PPS. Reachability judgements involving self-owned objects recruited a robust, right-lateralized parietal cluster, along with activations in the rIFG. The rIFG is frequently linked to the general recruitment of executive control^61,62^ including visuospatial working memory^63–65^ and early-stage inhibitory control^66,67^. These findings converge with prior reports of enhanced executive control in decisions involving self-owned objects within the PPS^29^. In contrast, reachability judgements involving other-owned objects elicited a more localized parietal response and were associated with increased activation in the pre-SMA. The pre-SMA is involved in the inhibition of motor responses^68–71^, and has been suggested to contribute to the endogenous, top-down suppression of inappropriate motor actions^72–74^. Consistent with this role, and in line with previous reports of slower and less accurate reachability judgement for other-owned objects in the PPS^30,99^, this activation may reflect an inhibitory mechanism that prevent actions inconsistent with social norms or personal goals, such as initiating movement towards objects belonging to others.

In contrast to PPS, reachability judgements for objects located in EPS did not evoke parietal activation, consistent with previous studies^49^. However, a key finding was that processing objects in EPS was associated with widespread engagement of the prefrontal cortex. While perception of stimuli in EPS, unlike in the PPS, is not typically marked by specific neural activations^49^, the proximity of objects to a virtual character in EPS may have heightened the weight attributed to the social context in which these objects are embedded. This, in turn, may have led to enhanced recruitment of prefrontal regions, which are known to support a range of higher-order processes, including self-referential cognition and social reasoning^76–79^, but also attention orientation and increased task demand^62^. Complementary contrasts against the implicit baseline (see Supplementary Information) provided additional information on the functional profile of EPS processing. While prefrontal activation emerged in the EPS > PPS contrast, it did not appear in the EPS > baseline comparison. A possibility, as discussed above, is that prefrontal regions are more strongly engaged during EPS judgments. In addition, prefrontal regions (considered a core node of the default mode network) have been shown to exhibit anticorrelations with parietal regions belonging to the dorsal attention and fronto-parietal control networks^80,81^. Accordingly, the strong fronto-parietal recruitment observed during PPS processing, particularly within parietal regions, may have reduced the relative contribution of prefrontal regions during near-space judgments. Altogether, these results show that this effect should be interpreted as a relative modulation in EPS with respect to PPS, rather than as evidence for processes that are exclusively engaged in EPS.

This social influence was further modulated by ownership: detailed contrasts between EPS and PPS for self- and other-owned objects revealed overlapping yet distinct activation profiles. Self-owned objects preferentially engaged a large prefrontal cluster, including both the vmPFC and dmPFC, while other-owned objects predominantly activated the vmPFC and the right posterior superior temporal sulcus (pSTS). The pSTS is a key region for the perception of social information^82,83^, and particularly for interpreting others’ actions and intentions^84–87^. Moreover, it has been proposed to play a role in linking contextual and perceptual information in social action-related scenarios^88^. Its preferential activation by other-owned objects near a virtual character may, therefore, reflect the integration of perceptual and inferential processes for interpreting the social significance of those items.

## Fronto-parietal recruitment as a function of ownership

To test the hypothesis that fronto-parietal activations are sensitive to object ownership, we conducted a targeted analysis within this network, revealing enhanced bilateral recruitment of parietal areas when participants evaluated self-owned stimuli in the PPS. The absence of preferential activation for other-owned objects further underscores the asymmetrical coding of ownership within this network, with a preferential engagement of the core PPS network in processing relevant targets. In contrast, in EPS, self-owned objects did not elicit significant activation, whereas other-owned objects selectively engaged the right parietal cortex. This region is recognized as part of the mirror neuron system^87^ and has previously been implicated as a key node within a “social brain network”, supporting action understanding and social coordination^75,89–91^. In the present study, when objects are self-owned and located within the participant’s immediate reach, they may be encoded primarily in relation to the participant’s own action affordances; in other words, they may be represented in terms of action relevance. On the contrary, when objects are other-owner (i.e., explicitly associated with another agent) and located beyond the participant’s immediate reach (i.e., in the other agent’s PPS), they may be encoded in relation to the other agent’s action affordances. This selective engagement suggests that ownership cues interact with spatial context to shape how action-related information is encoded across space, which lends support to recent models describing PPS as selectively tuned to integrate spatial and bodily information based on contextual cues, particularly when they hold relevance for potential interaction. Such context-dependent recruitment of the fronto-parietal network aligns with recent behavioral accounts highlighting the influence of social factors on PPS-related processing in relation to both objects affordances and social norms^47,99^.

## Object ownership in the medial prefrontal cortex

A central objective of the present study was to determine how self-relevant brain regions, specifically the vmPFC and dmPFC, encode object ownership in relation to spatial context. While univariate analyses yielded no significant ownership effect in the PPS, multivoxel pattern analysis (MVPA) uncovered finer-grained distinctions. The vmPFC discriminated self-owned objects above chance in the PPS, but failed to differentiate between self- and other-owned objects in EPS. This suggests that vmPFC activity does not reflect an inherent or fixed self-preference, but instead functions as a context-sensitive filter, tagging self-relevant stimuli when they are situationally or motivationally pertinent. This interpretation aligns with recent models that conceptualized the vmPFC not as an amodal “self-hub”, but as a dynamic, context-dependent filter of self-relevant information^79,92^.

In contrast, the dmPFC demonstrated consistent sensitivity to self-ownership across space, with accurate classification performance for self-owned objects in both PPS and EPS. This suggests that the dmPFC may support more enduring self-related information within a social context^93^, independently of immediate motivational salience^94^. Alternatively, or additionally, rather than encoding the “self” per se, the dmPFC could track the relational status of an object in a social context (“belonging to me” vs. “belonging to another”) and help maintain the distinction between items relevant to the self and those relevant to others. This interpretation aligns with a body of work linking the dmPFC to social comparison, self-other differentiation, and social inferential evaluation^95–98^. Under this view, the dmPFC may sustain ownership-related distinctions even when motivational relevance is low, as these distinctions are crucial for guiding social behavior and cooperation.

Overall, a key finding of the present study is that the engagement of the vmPFC in self-referential processing seems to be constrained by the spatial configuration of the environment in relation to action. This aligns with studies suggesting that transient disruption of the vmPFC does not typically abolish self-reference effects^100,116^. Notably, the vmPFC ROI in the present study largely corresponds to clusters identified in reverse-inference analysis by Lieberman et al.^79^, which primarily code for affective and situational processing. In contrast, the dmPFC ROI overlaps with clusters linked to self-related and social processing. These distinctions are further supported by recent evidence highlighting the role of the vmPFC in processing reward-related or motivational information, and of the dmPFC in tracking social information related to social conformity^100^. Taken together, these findings suggest that the vmPFC flexibly tags self-relevant stimuli with motivational value depending on context, while the dmPFC supports a sustained representation of self-related information within social contexts, particularly in relation to the role of object ownership in structuring appropriate social interaction. These results notably align with recent models of the mPFC that challenge the notion of a gradient from social to nonsocial cognition^77,98^, and rather describes a functional specialization where distinct subregions of the mPFC contribute to both social and cognitive processes^77,79^.

In terms of limitations, it should be noted that 22 usable datasets were obtained from the 25 participants initially tested, reflecting the maximum feasible sample given restricted access to the MRI scanner. Nonetheless, this sample size exceeds the a priori power estimate based on previous studies, which estimated that the recruitment of 17 participants would be sufficient (see Methods). It also outnumbers the sample sizes typically reported in earlier investigations of the neural correlates of reachability judgment tasks^49,104^ and falls within the range considered adequate for within-subject fMRI designs^101,102^. Although some authors have argued that approximately 27 participants would represent an optimal sample size for fMRI within-subject designs^103^, the present study can nonetheless be regarded as appropriately powered for the planned within-subject contrasts. Future replication with larger samples would, however, improve the precision and generalizability of the observed effects. In addition, future studies could extend the present design to clarify whether the mPFC responses observed here reflect sensitivity to motivational self-relevance, social-relational ownership, or their interaction. One possible approach would be to manipulate the presence or spatial location of the virtual character (e.g., removing it or repositioning it within the virtual environment). Such manipulations could help disentangle the effects associated with contextual factors inherent to the current setup, specifically those observed in the EPS. Furthermore, orthogonal manipulations of ownership and motivational value (e.g., by varying reward or valence independently of self/other attribution) would more precisely characterize the dissociation between vmPFC contributions to value-based self-relevance and dmPFC contributions to the social representation of ownership.

A further limitation relates to the fact that object ownership was experimentally assigned rather than grounded in real-world valuation or personal investment. While this approach ensures experimental control and likely reflects a transient or situational form of ownership, naturalistic ownership often carries motivational or affective significance that may recruit partially distinct neural mechanisms. Future work should therefore aim to disentangle these two facets of ownership: a transient, norm-based aspect reflecting socially permitted access and serving social coordination (e.g., the ownership of a glass of wine on a table in a restaurant), and a more identity-relevant or affective aspect (e.g., the ownership of a childhood toy or of one’s mobile phone). Such investigations could benefit from employing more immersive environments and interactive confederates, for instance through virtual reality setups or realistic interactive scenarios that better approximate everyday ownership and the social characteristics of the interacting agent.

## Conclusion

In sum, our findings demonstrate that the neural encoding of PPS and EPS is not uniform, but is modulated by the contextual factor of object ownership. Parietal regions traditionally associated with near-space and action-related processing were preferentially engaged when participants evaluated self-owned objects within PPS, whereas other-owned objects elicited a more restricted parietal response and recruited the pre-SMA. In EPS, ownership modulated the activity of prefrontal regions involved in social cognition and self-related processing, notably the vmPFC and dmPFC. Crucially, MPVA classification analyses revealed that vmPFC coding for self-ownership was contingent upon spatial proximity, reflecting a sensitivity to motivational relevance, whereas the dmPFC maintained a stable representation of self-ownership across both PPS and EPS. Together, these results challenge models that propose a unitary, centralized mechanism for self-ownership and, to a larger extent, self-relevance. Instead, they support a distributed context-sensitive framework in which motivational and social factors shape neural processing as a function of space.

## Method

### Participants

An a priori power analysis (G*Power version 3.1) indicated that 17 participants would be sufficient to achieve 80% power at an alpha level of 0.05, assuming a medium-to-large effect size (f = 0.3)^30^. However, in line with recommendations for within-subject fMRI designs suggesting optimal samples between 17 and 32 participants^101,102^, twenty-five right-handed healthy volunteers were recruited to ensure adequate statistical power and account for potential data loss (17 women, *M*_age_ = 21.98, *SD* = 4.04). The data of three participant were removed because of technical issues (1 participant) or excessive movement-induced noise (2 participants), computed as follows: if excluded volume > 4 minutes mean, if framewise displacement (FD) > 0.3 mm, or if more than 20% of FDs > 0.5 mm or > 1.5 times the derivative of the root mean square (RMS) variance (following the recommendations of Satterthwaite et al., 2013, and as implemented in fMRIPrep). Ultimately, the data of 22 participants were analyzed (14 female, *M*_age_ = 21.51, *SD*_age_ = 3.72). All participants had normal or corrected-to-normal vision, had no history of neurological or psychiatric disorder, and gave their formal written consent before taking part in the study. The experimental design was approved by the local ethics committee (reference CEICSH 114/2023), in accordance with The Code of Ethics of the World Medical Association (Declaration of Helsinki, 2013).

## Materials and procedure

**Fig. 6:**
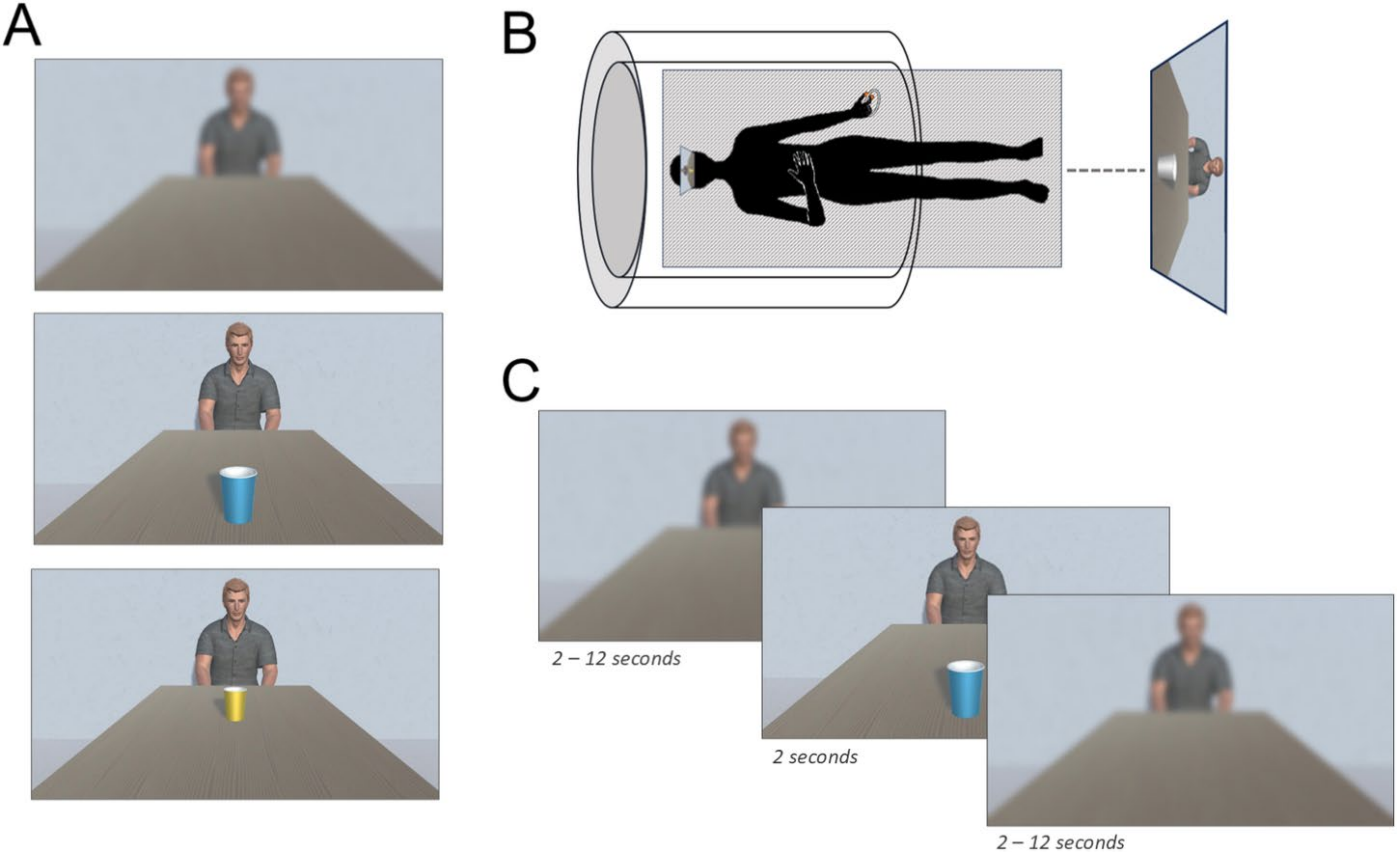
(**A**) Illustration of the virtual environment with the blurred mask (top panel), with a cup located in the participant’s peripersonal space (middle panel) or extrapersonal space (bottom panel). (**B**) Schematic representation of the experimental setup during the pre-experimental session. (**C**) Time-course of stimuli presentation.

Before entering the scanner, participants chose a colored paper cup among two (blue or yellow) in order to induce object ownership. They were told that the chosen paper cup was theirs and that they could keep it even after the experiment, and the non-chosen one belonged to a virtual character they would meet later. Both pre-experimental and experimental tasks used a virtual environment designed with Unity (version 2021.3.13f1) composed of a virtual table of 150 cm long with a 3D wood texture, at the extremity of which a neutral-faced virtual character taken from the ATHOS database^105^ was seated (Fig. 6 A). No spatial cue biasing the perception of distance or allowing for the development of strategies by participants was available in the virtual environment. The two paper cups were replicated in the virtual environment and designed to be isoluminant in Unity, with a luminance value of 100% (HSV) and maintained a comparable contrast ratio against the virtual table (yellow cup to table ratio: 1:6.36; blue cup to table ratio: 1:6.08). The virtual environment was displayed through a monitor screen placed in front of the MRI scanner visible through mirrors set at the MRI-head coil.

### Pre-experimental session

To account for inter-individual variability in the perception of distance in a virtual environment, participants performed a pre-experimental task where a neutral gray cup (i.e., with no ownership, Fig. 6 B) was randomly presented at 29 distances ranging from 5 to 145 cm from the proximal edge of the virtual table. For each trial, participants were asked to estimate if they would be able to reach the cup with their right hand without moving their shoulders or their trunk; no real movement was allowed. They provided their responses by pressing buttons on a response grip with their left index and thumb. At the end of this task, a reachability threshold was obtained by the computer for each participant using the formula:

$${\text{y }} = ({\text{exp }}(\alpha + \beta {\mathrm{x}}) )/({1} + {\text{ exp }}(\alpha + \beta {\mathrm{x}}) )$$

where x represents the distance of the cup on the table and y represents the participant’s probability of estimating the cup as “reachable”. The reachability threshold was obtained by computing -α/β and served afterwards to segment, for each participant, the table into a PPS and EPS region.

### Experimental session

In this task, a mask consisting of a gaussian blur was applied to the virtual environment with no cup on the table and presented for a jittered period of 2 to 12 s (Figure 6.C). Subsequently, one of the two cups (yellow or blue) was individually presented for 2 s either in the PPS (at −70%, −60%, or −50% from the previously computed reachability threshold), in the EPS (at +50%, +60%, and +70% from the previously computed reachability threshold) or, to enhance the complexity of the task, near the reachability threshold in 10% (22) of the trials, used as fillers (at -10%, 0% and +10% of the reachability threshold). Participants were instructed to imagine themselves seated at the table within the virtual environment, and to passively estimate whether self- and other-owned cups (yellow and blue) positioned at varying distances would be reachable with their right hand. They were explicitly instructed to make these reachability judgments solely from their own perspective and not from the viewpoint of the virtual character in front of them. To maintain engagement while minimizing the influence of motor preparation-related activations, participants were requested to respond only in 10% of the trials, specifically when a question mark appeared on the screen.

## fMRI data acquisition and pre-processing

All fMRI data were acquired using a 3T Siemens Magnetom Trio scanner (Siemens, Erlangen, Germany) and a 8-channel receive-only head coil equipped with a mirror. Blood oxygen level-dependent-sensitive (BOLD) fMRI images were acquired in an interleaved way through use of an T2* weighted echo-planar images (EPIs) (TR = 2000 ms, TE = 18 ms, FA = 85°, Field of View (FoV) = 200 mm × 200 mm, matrix size = 66 × 66, in-plane resolution = 3.03 mm × 3.03 mm) from 48 axial slices of 3-mm thickness with 3-mm interslice gap, covering the whole brain. A total of 730 volumes were acquired during the task. Structural T1-weighted images was collected (TR = 2400 ms, TE = 2.53 ms, FoV = 25.5 mm × 25.5 mm, matrix size = 256 × 256, in-plane resolution = 0.98 mm × 0.98 mm) and used to project the functional maps. Participants were instructed to refrain from moving to reduce movement artifacts, and head motion was minimized using ear pads.

Results included in this manuscript come from preprocessing performed using fMRIPrep 23.2.0^106^ (RRID:SCR_016216), which is based on Nipype 1.8.6^107^ (RRID:SCR_002502). Comprehensive description of the preprocessing procedure for both anatomical and functional imaging data can be found in the Supplementary Material.

## fMRI analysis

Preprocessed functional imaging data were analyzed using Statistical Parametric Mapping (SPM12, Wellcome Department of Imaging Neuroscience, London, UK) implemented in MATLAB R2024b (MathWorks Inc., Natick, MA, USA). For each participant, a first-level analysis was conducted using a General Linear Model (GLM) to estimate the hemodynamic response for each condition of interest. To optimize denoising while minimizing the risk of spurious signal influencing the results^108^, 36 regressors of non-interest resulting from 3D head motion estimation (x, y, z translation and three axis of rotation, their derivatives, quadratic terms, and squares of derivatives) to which were added *k* spike regressors (as proposed by Satterthwaite et al.^109^ and as implemented in fMRIPrep^106^) were added in the design matrix. A 2×2 factorial design was employed, with two factors each with two levels: Space (Peripersonal, Extrapersonal) and Ownership (Self, Other).

## Univariate analysis

We first conducted a whole-brain analysis to examine global neural activations in response to the experimental conditions. A *F*-contrast first assessed the interaction of Space and Ownership. Then, *t*-contrasts were computed to examine the main effects of Space (*PPS > EPS*, *EPS > PPS*) and Ownership (*Self > Other*, *Other > Self*). To get a better overview of the spatial neural processing of objects depending on their ownership, we computed *t-*contrasts comparing PPS and EPS for self- and other-owned objects (*PPS-Self > EPS-Self*; *EPS-Self > PPS-Self*; *PPS-Other > EPS-Other*; *EPS-Other > PPS-Other*). Individual *t-*contrast images were generated for each participant before being entered in a one-sample *t-*test for second-level analyses. Voxel significance was thresholded at p < 0.001 (uncorrected)^48^, with clusters considered as significant at p < 0.05 (FDR-corrected^110^).

To assess whether brain regions typically involved in processing stimuli in the PPS were modulated by object ownership, we computed additional *t-*contrasts comparing Self- and Other-owned objects within the PPS (*PPS-Self > PPS-Other*, *PPS-Other > PPS-Self*). Based on the well-established role of the fronto-parietal network in the PPS processing^13,50^, we selected regions of interest (ROIs) from Grivaz et al.^14^, including the postcentral and precentral gyri, supramarginal gyrus, and superior and inferior parietal lobules. Analyses were restricted to this core PPS network using the ROI analysis implemented in the WFU PickAtlas toolbox in SPM12. As studies have suggested that observing actions or objects related to a conspecific can activate observer’s fronto-parietal network^22,111,112^, we additionally investigated whether ownership-related effects extend near the virtual character by performing analogous contrasts in the EPS (*EPS-Self > EPS-Other*, *EPS-Other > EPS-Self*). Given our a priori hypotheses and the subtle nature of PPS-related sensorimotor activations^14,49^, we applied a more liberal statistical threshold (voxel-wise p < 0.005 uncorrected, cluster-level p < 0.05 FDR-corrected)^49^.

All coordinates are reported in MNI-152 space and anatomical labels were assigned using the Automated Anatomical Labeling (AAL) atlas, version 3v1^113^. When available, cytoarchitectonic location was derived from the JuBrain Anatomy Toolbox^114^. For regions not covered by the cytoarchitectonic maps, Brodmann area (BA) designations were provided.

## Multivariate analysis

Given the ongoing debate about the distinct roles of the dorsal and ventral mPFC in processing self- and other-related contextual information, we aimed to determine whether activation patterns in this region, if observed, were context-dependent. To examine this, and given that univariate studies frequently report overlapping activation in the mPFC for self- and other-related processing, we employed multivariate pattern analysis (MVPA) to investigate whether distinct multivoxel patterns could differentiate these processes. MVPA analyzes spatial patterns of neural activity and voxel relationships, enabling the detection of subtle, context-specific neural representations. Unlike methods that assess overall activation, MVPA thus captures fine-grained spatial patterns that reflect feature-selective neural populations. Regions of interest (ROIs) were defined a priori using the AAL atlas in the WFU PickAtlas toolbox in SPM12. Specifically, the ventral mPFC ROI was defined as a binary mask encompassing the orbital superior gyrus, the inferior frontal gyrus, and the gyrus rectus following Rolls et al.^113^, except for the ACC that is usually separated from the vmPFC in the literature about self-relevance^35^. The dorsal mPFC ROI was thus defined as the superior medial gyrus. MVPA was conducted using The Decoding Toolbox (v3.999^115^) in MATLAB, with beta images derived from the general linear model (GLM) on unsmoothed data. These beta-values were input into ROI-based MVPA classifiers, using a leave-one-subject-out (LOSO) cross-validation procedure to minimize overfitting and identify shared patterns across individuals. A linear support vector machine (SVM) classifier was trained on data from 21 participants, while the data from the remaining participant served as the test set. This LOSO cross-validation procedure was iteratively repeated such that each participant was used once as the test subject. Null confusion matrices were generated by randomly shuffling the pattern labels of the training and testing data within-subject 1000 times, and Bonferroni-corrected p-values were calculated based on the obtained distributions.

## Supplementary Information

Supplementary Information.

## Data Availability

The datasets analyzed during the current study are not publicly available due to data protection regulations and the nature of participants’ informed consent, but can be made available from the corresponding author upon reasonable request.
