# Supplementary material for: The contextual self: object ownership modulates neural encoding across peripersonal and extrapersonal spaces
Source: Sci Rep. 2026 Mar 24;16:14825. doi: 10.1038/s41598-026-44438-z (PMC13168643; doi:10.1038/s41598-026-44438-z)
Supplement: Supplementary file 1 — Supplementary Information. [file 41598_2026_44438_MOESM1_ESM.docx]

**Supplementary materials**

***Anatomical data preprocessing***

A total of 1 T1-weighted (T1w) images were found within the input BIDS dataset. The T1w image was corrected for intensity non-uniformity (INU) with N4BiasFieldCorrection^1^, distributed with ANTs 2.5.0^2^ (RRID:SCR_004757), and used as T1w-reference throughout the workflow. The T1w-reference was then skull-stripped with a Nipype implementation of the antsBrainExtraction.sh workflow (from ANTs), using OASIS30ANTs as target template. Brain tissue segmentation of cerebrospinal fluid (CSF), white-matter (WM) and gray-matter (GM) was performed on the brain-extracted T1w using fast^3^ (FSL (version unknown), RRID:SCR_002823). Brain surfaces were reconstructed using recon-all^4^ (FreeSurfer 7.3.2, RRID:SCR_001847), and the brain mask estimated previously was refined with a custom variation of the method to reconcile ANTs-derived and FreeSurfer-derived segmentations of the cortical gray-matter of Mindboggle^5^ (RRID:SCR_002438). Volume-based spatial normalization to one standard space (MNI152NLin2009cAsym) was performed through nonlinear registration with antsRegistration (ANTs 2.5.0), using brain-extracted versions of both T1w reference and the T1w template. The following template was were selected for spatial normalization and accessed with TemplateFlow (23.1.0, Ciric et al. 2022): ICBM 152 Nonlinear Asymmetrical template version 2009c^6^ [ RRID:SCR_008796; TemplateFlow ID: MNI152NLin2009cAsym].

***Functional data preprocessing***

For each BOLD run found per subject, the following preprocessing was performed. First, a reference volume was generated, using a custom methodology of fMRIPrep^7^, a Nipype-based tool^8,9^ for use in head motion correction. Head-motion parameters with respect to the BOLD reference (transformation matrices, and six corresponding rotation and translation parameters) are estimated before any spatiotemporal filtering using mcflirt^10^ (FSL). The BOLD reference was then co-registered to the T1w reference using bbregister (FreeSurfer) which implements boundary-based registration^11^. Co-registration was configured with six degrees of freedom. Several confounding time-series were calculated based on the preprocessed BOLD: framewise displacement (FD), DVARS and three region-wise global signals. FD was computed using two formulations following Power (absolute sum of relative motions^12^) and Jenkinson^10^ (relative root mean square displacement between affines). FD and DVARS are calculated for each functional run, both using their implementations in Nipype (following the definitions by Power et al.^12^). The three global signals are extracted within the CSF, the WM, and the whole-brain masks. Additionally, a set of physiological regressors were extracted to allow for component-based noise correction (CompCor^13^). Principal components are estimated after high-pass filtering the preprocessed BOLD time-series (using a discrete cosine filter with 128s cut-off) for the two CompCor variants: temporal (tCompCor) and anatomical (aCompCor). tCompCor components are then calculated from the top 2% variable voxels within the brain mask. For aCompCor, three probabilistic masks (CSF, WM and combined CSF+WM) are generated in anatomical space. The implementation differs from that of Behzadi et al^10^. in that instead of eroding the masks by 2 pixels on BOLD space, a mask of pixels that likely contain a volume fraction of GM is subtracted from the aCompCor masks. This mask is obtained by dilating a GM mask extracted from the FreeSurfer’s aseg segmentation, and it ensures components are not extracted from voxels containing a minimal fraction of GM. Finally, these masks are resampled into BOLD space and binarized by thresholding at 0.99 (as in the original implementation). Components are also calculated separately within the WM and CSF masks. For each CompCor decomposition, the k components with the largest singular values are retained, such that the retained components’ time series are sufficient to explain 50 percent of variance across the nuisance mask (CSF, WM, combined, or temporal). The remaining components are dropped from consideration. The head-motion estimates calculated in the correction step were also placed within the corresponding confounds file. The confound time series derived from head motion estimates and global signals were expanded with the inclusion of temporal derivatives and quadratic terms for each^14^. Frames that exceeded a threshold of 0.5 mm FD or 1.5 standardized DVARS were annotated as motion outliers. Additional nuisance timeseries are calculated by means of principal components analysis of the signal found within a thin band (crown) of voxels around the edge of the brain, as proposed by Patriat, Reynolds, and Birn^15^. All resamplings can be performed with a single interpolation step by composing all the pertinent transformations (i.e. head-motion transform matrices, susceptibility distortion correction when available, and co-registrations to anatomical and output spaces). Gridded (volumetric) resamplings were performed using nitransforms, configured with cubic B-spline interpolation. All functional volumes were smoothed with a Gaussian kernel with FWHM of 8 × 8 × 8 mm.

Many internal operations of FMRIPREP use Nilearn^16^ [ RRID:SCR_001362], principally within the BOLD-processing workflow. For more details of the pipeline see <https://fmriprep.readthedocs.io/en/20.2.0/workflows.html>.

**References**

1. Tustison, N. J. et al. N4ITK: Improved N3 bias correction. IEEE Trans. Med. Imaging 29, 1310–1320 (2010). https://doi.org/10.1109/TMI.2010.2046908

2. Avants, B. B., Epstein, C. L., Grossman, M. & Gee, J. C. Symmetric diffeomorphic image registration with cross-correlation: Evaluating automated labeling of elderly and neurodegenerative brain. Med. Image Anal. 12, 26–41 (2008). https://doi.org/10.1016/j.media.2007.06.004

3. Zhang, Y., Brady, M. & Smith, S. Segmentation of brain MR images through a hidden Markov random field model and the expectation-maximization algorithm. IEEE Trans. Med. Imaging 20, 45–57 (2001). https://doi.org/10.1109/42.906424

4. Dale, A. M., Fischl, B. & Sereno, M. I. Cortical surface-based analysis: I. Segmentation and surface reconstruction. NeuroImage 9, 179–194 (1999). https://doi.org/10.1006/nimg.1998.0395

5. Klein, A. et al. Mindboggling morphometry of human brains. PLoS Comput. Biol. 13, e1005350 (2017). https://doi.org/10.1371/journal.pcbi.1005350

6. Fonov, V. S., Evans, A. C., McKinstry, R. C., Almli, C. R. & Collins, D. L. Unbiased nonlinear average age-appropriate brain templates from birth to adulthood. NeuroImage 47 (Suppl. 1), S102 (2009). https://doi.org/10.1016/S1053-8119(09)70884-5

7. Esteban, O. et al. fMRIPrep: A robust preprocessing pipeline for functional MRI. Nat. Methods

16, 111–116 (2019). https://doi.org/10.1038/s41592-018-0235-4

8. Gorgolewski, K. et al. Nipype: A flexible, lightweight and extensible neuroimaging data processing framework in Python. Front. Neuroinform. 5, 13 (2011). https://doi.org/10.3389/fninf.2011.00013

9. Gorgolewski, K. J. et al. Nipype (software) (2018). https://doi.org/10.5281/zenodo.596855

10. Jenkinson, M., Bannister, P., Brady, M. & Smith, S. Improved optimization for the robust and accurate linear registration and motion correction of brain images. NeuroImage 17, 825–841 (2002). https://doi.org/10.1006/nimg.2002.1132

11. Greve, D. N. & Fischl, B. Accurate and robust brain image alignment using boundary-based registration. NeuroImage 48, 63–72 (2009). https://doi.org/10.1016/j.neuroimage.2009.06.060

12. Power, J. D. et al. Methods to detect, characterize, and remove motion artifact in resting-state fMRI. NeuroImage 84, 320–341 (2014). https://doi.org/10.1016/j.neuroimage.2013.08.048

13. Behzadi, Y., Restom, K., Liau, J. & Liu, T. T. A component-based noise correction method (CompCor) for BOLD and perfusion-based fMRI. NeuroImage 37, 90–101 (2007). https://doi.org/10.1016/j.neuroimage.2007.04.042

14. Satterthwaite, T. D. et al. An improved framework for confound regression and filtering for control of motion artifact in the preprocessing of resting-state functional connectivity data. NeuroImage 64, 240–256 (2013). https://doi.org/10.1016/j.neuroimage.2012.08.052

15. Patriat, R., Reynolds, R. C. & Birn, R. M. An improved model of motion-related signal changes in fMRI. NeuroImage 144, 74–82 (2017). https://doi.org/10.1016/j.neuroimage.2016.08.051

16. Abraham, A. et al. Machine learning for neuroimaging with scikit-learn. Front. Neuroinform. 8, 14 (2014). https://doi.org/10.3389/fninf.2014.00014

***Supplementary results***

*PPS vs. baseline.*

Table S1. Brain regions showing significant activations for the contrast between peripersonal space and the implicit baseline. x, y, z = peak coordinates (MNI); T = t-statistic.

|  | | | Peak-level | | | | | Cluster-level | | |
| --- | --- | --- | --- | --- | --- | --- | --- | --- | --- | --- |
| Macroanatomical location | | Microanatomical composition | x | y | z | *T* | *P* | Number of voxels | *P _uncorrected_* | *P _FDR_* |
| ***PPS > Baseline*** | | | | | | | | | | |
|  | Left supplementary motor area, Right supplementary motor area | Area 6mr / preSMA (41.4%) | 1  7  4 | -2  4  13 | 60  57  48 | 6.93  5.37  4.94 | 0  0  0 | 121 | 0.000 | 0.000 |
|  | Right inferior parietal lobule,  Postcentral gyrus,  Superior parietal lobule | hIPS3(IPS) (17.5%),  hIP2 (IPS) (16.1%),  Area 2 (14.4%),  Area 1 (10.2%) | 49  40  43 | -41  -53  -32 | 57  54  45 | 6.09  5.55  5.07 | 0  0  0 | 161 | 0.000 | 0.000 |
|  | Right superior parietal lobule,  Precuneus, Cuneus | Area 7P (SPL) (67.2%) | 13  13 | -77  -68 | 54  51 | 5.47  4.07 | 0  0 | 19 | 0.006 | 0.015 |
|  | Left precentral gyrus,  Superior frontal gyrus | Area 6d1 (27.4%),  Area 6d3 (12.6%) | -29 | -8 | 54 | 5.27 | 0 | 15 | 0.012 | 0.028 |
|  | Left inferior parietal lobule,  Postcentral gyrus | hIP3 (IPS) (28.7%),  Area 7A (SPL) (18.7%),  hIP2 (IPS) (16.4%),  hIPS1 (IPS) (14.8%) | -44  -32  -32 | -38  -56  -47 | 45  51  42 | 5.27  5.12  4.72 | 0  0  0 | 108 | 0.000 | 0.000 |
|  | Right precentral gyrus,  Postcentral gyrus | Area 4p (38.9%) | 34  31  34 | -20  -14  -20 | 57  66  45 | 4.89  4.25  3.92 | 0  0  0 | 24 | 0.002 | 0.008 |
|  | Right precentral gyrus | BA6, BA8 | 55 | 7 | 42 | 4.70 | 0 | 12 | 0.022 | 0.044 |

*EPS vs. baseline.*

Table S2. Brain regions showing significant activations for the contrast between extrapersonal space and the implicit baseline. x, y, z = peak coordinates (MNI); T = t-statistic.

|  | | | Peak-level | | | | | Cluster-level | | |
| --- | --- | --- | --- | --- | --- | --- | --- | --- | --- | --- |
| Macroanatomical location | | Microanatomical composition | x | y | z | *T* | *P* | Number of voxels | *P _uncorrected_* | *P _FDR_* |
| ***EPS > Baseline*** | | | | | | | | | | |
|  | Left supplementary motor area,  Right supplementary motor area | Area 6mr (preSMA) (28.6%),  Area 6mc (SMA) (12.3%) | -2  -2  1 | -8  4  -5 | 66  57  51 | 5.48  5.30  4.21 | 0  0  0 | 64 | 0.000 | 0.000 |
